# Genetic diversity and divergence at the *Arbutus unedo* L. (Ericaceae) westernmost distribution limit

**DOI:** 10.1371/journal.pone.0175239

**Published:** 2017-04-06

**Authors:** Maria Margarida Ribeiro, Andrea Piotti, Alexandra Ricardo, Daniel Gaspar, Rita Costa, Laura Parducci, Giovanni Giuseppe Vendramin

**Affiliations:** 1 Departamento de Recursos Naturais e Desenvolvimento Sustentável, Instituto Politécnico de Castelo Branco, Escola Superior Agrária, Castelo Branco, Portugal; 2 Plant Ecology/ Department of Ecology and Genetics, Evolutionary Biology Centre, Uppsala University, Norbyvägen 18 D, Uppsala, Sweden; 3 Forest Research Centre, School of Agriculture, University of Lisbon, Tapada da Ajuda, Lisbon, Portugal; 4 Institute of Biosciences and BioResources, National Research Council, Via Madonna del Piano 10, Sesto Fiorentino (Firenze), Italy; 5 INIAV, Instituto Nacional de Investigação Agrária e Veterinária, I.P., Av. República, Quinta do Marquês Oeiras, Portugal; National Cheng Kung University, TAIWAN

## Abstract

Mediterranean forests are fragile ecosystems vulnerable to recent global warming and reduction of precipitation, and a long-term negative effect is expected on vegetation with increasing drought and in areas burnt by fires. We investigated the spatial distribution of genetic variation of *Arbutus unedo* in the western Iberia Peninsula, using plastid markers with conservation and provenance regions design purposes. This species is currently undergoing an intense domestication process in the region, and, like other species, is increasingly under the threat from climate change, habitat fragmentation and wildfires. We sampled 451 trees from 15 natural populations from different ecological conditions spanning the whole species’ distribution range in the region. We applied Bayesian analysis and identified four clusters (north, centre, south, and a single-population cluster). Hierarchical AMOVA showed higher differentiation among clusters than among populations within clusters. The relatively low within-clusters differentiation can be explained by a common postglacial history of nearby populations. The genetic structure found, supported by the few available palaeobotanical records, cannot exclude the hypothesis of two independent *A*. *unedo* refugia in western Iberia Peninsula during the Last Glacial Maximum. Based on the results we recommend a conservation strategy by selecting populations for conservation based on their allelic richness and diversity and careful seed transfer consistent with current species’ genetic structure.

## Introduction

The contemporary genetic structure of a species offers important information about its responses to past geological and climatic events, which had often played a crucial role in shaping the current distribution range. The spatial distribution of genetic variation is strongly influenced by neutral and adaptive processes and, at the population level, the amount of extant genetic variation plays a key role in maintaining the adaptive potential to face future environmental changes [1]. Among other factors, the lack of genetic connectivity between populations can accelerate the within-population genetic loss in woody plants [2]. In the Mediterranean basin, geographical isolation at the biogeographical scale was mainly promoted by Pleistocene climatic oscillations, which determined the presence of refugial areas often hosting different genetic lineages of plant species [3]. The Iberian Peninsula (IP) is one of the major Pleistocene glacial refugial areas for European species, where multiple refugia exist [4,5]. Mediterranean forests growing on the IP are fragile ecosystems currently threatened by human-related activities like grazing, fires and intensive forest cutting. This region is also particularly vulnerable to recent global warming and reduction of precipitation, and simulation studies show a long-term negative effect on vegetation due to increase in drought and burnt area by fires [6].

The evergreen *Arbutus unedo* L. (strawberry tree) is a small tree native to the Mediterranean region found in western, central, and southern Europe, north-eastern Africa, Canary Islands and western Asia. The species distribution is mostly typical of Mediterranean sclerophyllous and laurel-like vegetation, mainly within coastal and inland areas, where frost or summer dryness are not too intense [7]. *Arbutus unedo* genetic structure has been investigated at both local and biogeographical scale using different types of markers [5,8,9,10,11,12,13]. In Portugal, strawberry tree has a widespread distribution strongly related to soil characteristics and climate, as well as to landscape fragmentation, wildfires, and domestication [14]. *Arbutus unedo* fruit has strong antioxidant properties and it can be consumed fresh or processed, and it is used in Portugal to produce the liquor ‘aguardente’, an important source of income for land users, which has led to an on-going process of domestication of the species through clonal propagation of genotypes selected for fruit quality [15] and to the use of seeds from unknown origin. This species has economic importance to be used in Portugal and southern Europe, with different commercial uses from processed and fresh fruit production to ornamental, pharmaceutical and chemical industrial applications, due to the phenolic acids and terpenoid compounds with strong antioxidant activity, vitamin C and tannin content [15 and references therein, 16, 17]. Nevertheless, this species remains largely underutilized, and organizations such as FAO [18] developed efforts to increase the use of those species.

Anthropogenic impacts on forest genetic resources include practices and processes that may affect the genetic layout of natural populations, including: (i) breeding and selection, (ii) seed transfer and (iii) environment alterations [19]. The effect of wildfires can greatly impact the distribution of genetic variation at regional scale, since a likely outcome of repeated wildfires is the effective population size reduction and the loss of within-population genetic diversity [20]. Regions with more severe droughts might also be affected by an increase of wildfire activity and intensity, which may have large impacts on vegetation density and distribution. In the Mediterranean basin, for instance, the area burned by wildfire is expected to increase by a factor of 3 to 5 by the end of the XXI century [21]. Nowadays, the total number of wildfires and the percentage of burned areas in Portugal are already considerably higher than in other southern Mediterranean countries, with figures showing a strong tendency to increase in the next years (S1 Fig). This is likely due to a decrease in agricultural areas in favour of woodlands [22]. Shift in land use coupled with growth of tree plantations (mainly dense coniferous stands) increased fire regularity, resulting in large and catastrophic wildfires, which in the last few decades have resulted in a marked rise of the areas burnt annually in Portugal [23].

The ongoing domestication process, together with the increasing frequency of wildfires due to higher temperatures and lower precipitations, demands further knowledge of the *A*. *unedo* genetic diversity at the regional scale to build dynamic conservation programs in Portugal [24 and references therein]. Due to the lack of information about the levels and distribution of genetic diversity at the regional scale, we sampled 15 natural populations throughout the westernmost part of the IP, and genotyped 451 individuals using chloroplast microsatellite markers (cpSSRs). The chloroplast genome is maternally inherited in the strawberry tree (our unpublished data), reflecting seed flow only. Moreover, this genome has a smaller effective population size, which makes it more susceptible to genetic drift and population or species differentiation [25]. Our specific aims were to: i) investigate the spatial distribution of genetic variation of *A*. *unedo* in Portugal, and to ii) provide recommendations for species’ conservation and help in provenance region design.

## Material and methods

### Study species

*Arbutus unedo* is an evergreen species, a shrub or a small tree up to 8–10 m. The fruit is a round berry (ca. 20 mm wide) and bright red when ripe in autumn. The berries with a fleshy pulp are mainly eaten by seed-disperser birds, which represent the main *A*. *unedo* seed dispersal vector [26,27]. The inflorescence is a panicle which appears in autumn, the flowers are insect pollinated [28 and our unpublished data], and crop fruit production starts when the plants are about six to seven years old (F. Gomes, personal communication). The *A*. *unedo* plants can easily resprout and survive if damaged (e.g., by fire or grazing) [29]. This species is a marginal woodland plant, unable to endure shading and to successfully compete for light [26]. *Arbutus unedo* prefers siliceous or decarbonated substrata and can grow on alkaline and relatively acidic soils, but grows satisfactorily under a very wide range of soil conditions, with pH varying from 4 to 7, excluding waterlogged soils [26].

### Sample collection

The leaves for DNA extraction were collected in ca. 30 individuals from 15 natural populations sampled across the species’ natural range in Portugal (Table 1, S2 Fig). Samples were stored at -80°C until DNA extraction. Sampled trees were separated by a minimum distance of ca. 30 m to avoid collecting related individuals and the coordinates for each individual were obtained with a GPS receiver. Our sampling took into account the environmental diversity in different soil and climate types [30,31] in Portugal (Table 1).

**Table 1:** Name, code, geographic location, soil and climate types of the sampled populations.

Population name	Code	Longitude^a^ W	Latitude^a^ N	Altitude (m a.s.l.)	Soil type	Climate type
Peneda-Gerês	PG	-8.18	41.77	611	Humic Cambisol	Tem. Oceanic
Chaves	CH	-7.44	41.71	470	Humic Cambisol	Med. Pluviseasonal Oceanic
Bragança	B	-6.95	41.5	623	Lithosol	Med. Pluviseasonal Oceanic
Gardunha	G	-7.41	40.12	728	Dystric Cambisol	Med. Pluviseasonal Oceanic
Alvoco	AV	-7.84	40.28	395	Humic Cambisol	Med. Pluviseasonal Oceanic
Oleiros Norte	ON	-7.77	39.96	678	Lithosol	Med. Pluviseasonal Oceanic
Pampilhosa da Serra	PS	-8.05	40	538	Humic Cambisol	Med. Pluviseasonal Oceanic
Sertã-Figueiredo	SF	-7.99	39.85	465	Humic Cambisol	Med. Pluviseasonal Oceanic
Arrábida	A	-9.01	38.47	252	Orthic Podzol	Med. Pluviseasonal Oceanic
Viseu	V	-7.83	40.8	641	Humic Cambisol	Med. Pluviseasonal Oceanic
São Mamede	SM	-7.41	39.4	681	Dystric Cambisol	Med. Pluviseasonal Oceanic
Herdade Parra	HP	-8.42	37.31	165	Lithosol	Med. Pluviseasonal Oceanic
Barranco do Velho	BV	-7.95	37.25	489	Lithosol	Med. Pluviseasonal Oceanic
Monchique	M	-8.52	37.33	369	Orthic Luvisol	Med. Pluviseasonal Oceanic
Espinhaço Cão	EC	-7.98	37.18	225	Chromic Luvisol	Med. Pluviseasonal Oceanic

^**a**^ decimals degrees

### Ethics statement

Leaf sampling of *A*. *unedo* adult trees was non-destructive. No specific permission was required to sample the species in Portugal. Field studies did not involve endangered or protected species.

### DNA extraction, amplification and sequencing

We extracted total DNA from frozen leaves with a Dneasy® Plant Mini kit (QIAGEN, Hilden, Germany), following the manufacturer’s instructions. Six chloroplast microsatellite primers (Table 2) were designed based on the chloroplast genome [32]. The genome was scanned for cpSSRs loci using the software SciroKo 3.4 [33]. Primers were designed in the flanking regions of the detected SSR repeats using Primer3 v. 0.4.0 [34]. The primer design parameters used were: i) primer length (we selected 18–23 bp, with 20 bp being optimal), ii) PCR product sizes (100–450 bp, by using 60°C as an optimum melting temperature of the PCR primers), and iii) GC > 40%. Each forward primer was fluorescently labelled with 6-FAM dye (AB PRISM Primer Applied Biosystems). All the individuals were genotyped with the four polymorphic markers: AU1, AU2, AU4, and AU7.

**Table 2:** Code, repeat unit, size, fragment start and end and position relative to the start of the fragment of the six *A*. *unedo* cpSSR primers used in this study.

Code	Repeat Unit	Size (bp)	Frag. start	Frag. end	Position	Primers
AU1	(A)15	312	4164	4475	80	CCGCACTTAAAAGCCGAGTA
						GGTAAATGGTGGGTTCGTTG
AU2	(T)18	294	75426	75719	24	CCAAGCATTCCCTGAAAAGA
						GGATTTGAACCTGCGACATT
AU4	(AT)5	387	21190	21571	184	TCTCCCAATTCGACCAAGTC
						AGGGGTTGTGGATACTGCTG
AU5	(AT)5	311	51000	51310	166	TGGCCAACTCTACCAAATCC
						TCCCCCAAATGCAAATTAAA
AU6	(GA)5	151	56699	56849	45	CCCTCTTGCCAGATTTATCG
						TTTTCGGGAGTTGTTTGTCC
AU7	(TA)5	376	13771	14146	119	CCAATAGCCCAAAGAAACGA
						TGCCCTAGACCTGTCCTTTG

Two multiplex PCR were performed using the Type-it® Microsatellite PCR Kit (Qiagen). Forward and reverse primers for each locus were mixed at equal proportion and used in the multiplex PCR reactions. The first multiplex mix (with AU2 and AU7) was prepared in a 6 μL final reaction volume containing 1 μL of DNA (~10 ng), 3 μL of 2× Type-it mix, 1.4 μL dH2O and 0.6 μL of primer pre-mix 1 (0.1 μM each primer). The second mix (with AU1 and AU4) contained the same amount of total volume, DNA and master mix and 2 μL of primer pre-mix 2 (0.1 μM each AU1 primer and 0.2 μM each AU4 primer). The two multiplexes were amplified with the same PCR profile: an initial activation step of 5 min at 95°C, followed by 30 cycles of 30 s denaturation at 95°C, 90 s annealing at 57°C and 30 s extension at 72°C, concluding with a final extension for 30 min at 60°C. The amplified products were mixed with Hi-Di™ Formamide (Applied Biosystems) and GeneScan™ 500 LIZ™ dye size standard (Applied Biosystems) according to standard protocols and separated on an AB 3500 DNA genetic analyzer (Applied Biosystems). The molecular sizes of the fragments were binned using the GeneMarker® v2.4.0 software.

### Data analysis

#### Genetic diversity

The four cpSSR fragments analysed were combined in order to derive the chloroplast haplotype of each individual. An haplotype network, a minimum spanning tree based on the differences in number of repeats between haplotypes, was created using the mst function and visualized through the plot.mst function in the *pegas* package in R [35,36]. The number of haplotypes (N_h_), the unbiased haplotypic diversity based on haplotype frequencies (H_e_) [37] and the haplotypic richness A_R_ (number of different haplotypes found when a specific sample size is sampled from that population—in our study, the population size was fixed at 30), were computed for each population using the program CONTRIB v. 1.4 [38]. The effective number of haplotypes (A_e_) and the number of private haplotypes (Ph) were calculated in GenAlEx v. 6.501 [39]. CONTRIB also provided a measure of the contribution of each population to the total diversity (CT%) and the total haplotypic richness (CTR%). In both cases, the contributions were split in a first component, due to population diversity, and a second component, due to the differentiation from the remaining populations [38]. Additionally, the average Goldstein genetic distance among individuals within each population was computed according to the stepwise mutation model (SMM) estimator D^2^_sh,_ [40], as modified for fully linked markers [41]. D^2^_sh_ was estimated on the original data and on a transformed dataset (ng = no gaps), where the gap between alleles were eliminated to mitigate the possible impact of indels, by in-house R scripts. The relationship between the estimates of diversity (N_h_ and H_e_) and the genetic distances among individuals within populations D^2^_sh_ and D^2^_sh_^ng^ was tested with Pearson's product-moment correlation in R.

#### Genetic and phylogeographic structure

Bayesian analysis using a spatial clustering model implemented in BAPS 5.4 [42] was used to infer population genetic structure. The use of spatial information increases the power to correctly detect the underlying population structure [43]. The analysis was repeated 10 times for each *K* (from one to 15) to obtain the optimal number of clusters. The best partition of populations into *K* clusters was identified as the one with the highest log marginal likelihood.

The extent of the genetic structure was further explored using a non-hierarchical and hierarchical analysis of molecular variance (AMOVA) implemented in the Arlequin 3.5 software [44]. We used the infinite allele mutation model (IAM) considering the distances between haplotypes as the number of different alleles, and the stepwise mutation model (SMM) considering those distances as the number of repeat units for each locus. To perform the SMM analysis, we coded cpSSR data in a binary way, as in Heuertz *et al*. [45]. Overall, this test estimated the variance components among and within populations. The hierarchical AMOVA (based on BAPS clustering) estimated the partition of the genetic variation among clusters, among populations within clusters and within populations. The significance values were computed by 1,000 permutations.

We checked for the presence of a phylogeographic structure by comparing differentiation of unordered alleles (G_ST_) and ordered alleles (N_ST_) with the PermutCpSSR 1.2.1 software [46]. N_ST_ is defined as a measure of genetic differentiation among populations when the distance between haplotypes is taken into account, whereas G_ST_ is a measure of genetic differentiation that makes use only of haplotypic frequencies. The significance of the two differentiation statistics was tested with 10,000 random permutations of individuals among populations. To test if the observed N_ST_ value is larger than the G_ST_, we counted how many permutated N_ST_ values were larger than the observed N_ST_. Statistical significance may indicate the presence of a phylogeographic structure [46], i.e., closely related haplotypes are more often found in the same population than would be expected by chance. Finally, isolation by distance (IBD) was tested comparing genetic and log-transformed geographic distances between population pairs, using F_ST_/(1-F_ST_) as genetic distance. The statistical significance of the Mantel test correlation coefficient was obtained from 1,000 permutations using Arlequin 3.5.

## Results

### Genetic diversity

**Fig 1:**
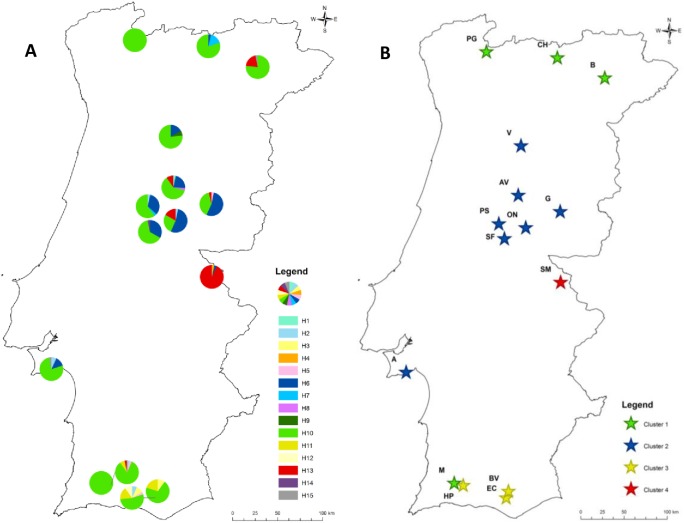
Haplotypes distribution and Bayesian analysis results. (A) Distribution of the haplotypes in each population. (B) Spatial clustering defined in the BAPS analysis. The best partition displayed 4 clusters: northern, southern and central clusters, and an outlier cluster, the SM population. Cluster 1: [M, PG, CH, B; red star], cluster 2: [HP, BV, EC; green star]; cluster 3: [A, SF, ON, G, AV, PS, V; blue star] and cluster 4: [SM, yellow star]. See Table 1 for population abbreviations.

**Fig 2:**
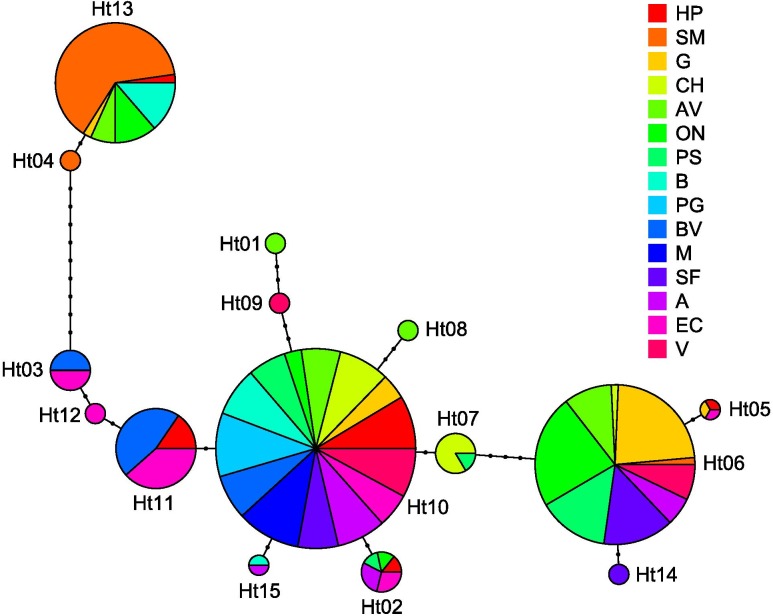
Haplotype network. The haplotype network is a minimum spanning tree based on the differences in number of repeats. The circles size is proportional to rescaled haplotype frequencies (see M&M for details). Colours indicate in which populations is present each haplotype. In case one haplotype is present in several populations, the circle is divided in circle sectors that are proportional to the number of plants in each population having that haplotype.

We obtained 15 haplotypes after genotyping 451 individuals with four polymorphic cpSSRs (S1 Table). With only one exception (i.e. population SM), the most frequent haplotype is H10 (Figs 1A and 2). The population SM differed from the others by showing haplotype H13 at high frequency (> 0.90) (Figs 1A and 2). Five populations showed private haplotypes [AV (H8, H1), SM (H4), V (H9), SF (H14), EC (H12)].

The average haplotypic diversity within populations was 0.40 (Table 3), with populations EC and ON displaying the highest haplotypic diversity (H_e_) and effective number of haplotypes (A_e_). The population EC showed also the highest values for N_h_ (6). Other populations from the central region (G, AV and PS), had H_e_ values over 0.50 and A_e_ > 2. The populations PG and M were fixed for the most common haplotype and also the population SM showed a very low value of diversity (0.13).

**Table 3:** Main within-population genetic parameters for the 15 populations analysed in this study. N = population sample size; N_h_ = number of haplotypes; A_e_ = effective number of haplotypes; A_R_ = haplotypic richness; H_e_ = unbiased haplotype diversity with standard errors in brackets; D^2^_sh_ is the average genetic distances among individuals and D^2^_sh_^ng^ is the average genetic distances among individuals with no gaps; Ph = number of private haplotypes.

Region	Pop	N	N_h_	A_e_	A_R_	H_e_	D^2^_sh_	D^2^_sh_^ng^	Ph
	PG	30	1	1	0	0.00 (0.00)	0	0	0
North	CH	30	3	1.5	2	0.34 (0.10)	0.32	0.13	0
	B	30	3	1.59	2	0.38 (0.09)	10.11	0.78	0
	G	30	4	2.24	3	0.57 (0.05)	5	0.83	0
	AV	31	5	2.28	3.9	0.58 (0.08)	8.52	1.11	2
	ON	30	4	2.6	3	0.64 (0.06)	14.95	1.78	0
Centre	PS	30	4	2.11	3	0.54 (0.07)	1.87	0.49	0
	SF	30	3	1.95	2	0.50 (0.06)	2.03	0.54	1
	A	30	4	1.64	3	0.40 (0.10)	1.06	0.32	0
	V	30	3	1.61	2	0.39 (0.10)	1.37	0.34	1
	SM	30	3	1.15	2	0.13 (0.08)	3.78	0.44	1
	HP	30	5	1.42	4	0.31 (0.11)	2.55	0.37	0
South	BV	30	3	1.85	2	0.48 (0.09)	0.44	0.44	0
	M	30	1	1	0	0.00 (0.00)	0	0	0
	EC	30	6	2.96	5	0.69 (0.08)	1.11	0.79	1
	Mean	30.07	3.47	1.79	2.46	0.4	3.54	0.56	0.4
	SE	0.07	0.35	0.15	1.35	0.06	4.38	0.46	

We found no correlation between D^2^_sh_ and genetic diversity estimates (Table 3). The populations with high values of D^2^_sh_, such as the B population, did not display high values of genetic diversity (N_h_ and H_e_). However, if we consider the genetic distance with no gaps (D^2^_sh_^ng^), a significant correlation was found between the average genetic distance and the estimates of genetic diversity (N_h_ and H_e_) (Table 3). This discrepancy was probably due to the presence of an insertion determining a 9 bp gap between alleles 285 and 294 at locus AU2 (see S1 Table).

**Fig 3:**
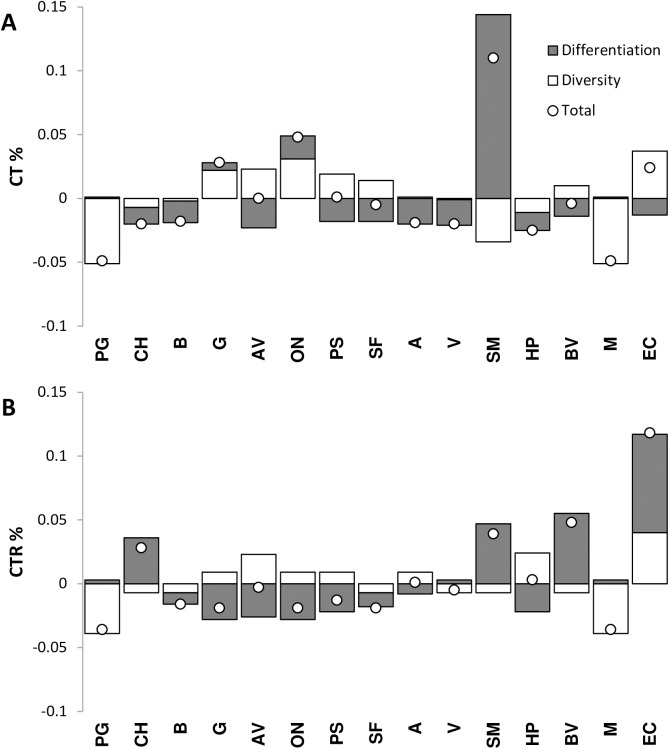
Population diversity partition according to Petit et al. [38] (A) Contribution to the total diversity (CT%) of each population subdivided into own-diversity (white) and divergence (grey) components. (B) Contribution to the total haplotypic richness (CTR%) of each population subdivided into own-diversity (grey) and divergence (white) components.

The population SM showed the highest contribution to the differentiation of populations, while populations G and ON showed the highest contribution to the total diversity. The populations that gave the highest contribution to the population diversity were AV, ON and EC. The populations PG and M contributed negatively to both components of the total diversity (diversity and divergence components), showing only one haplotype (CT%, Fig 3A). The population EC had the highest contribution to the total allelic richness, followed by populations SM and BV (CTR%, Fig 3B).

### Population differentiation and Bayesian cluster analysis

The Bayesian clustering suggests the presence of four major groups of populations: a northern cluster, a central cluster with populations from the coast and inland, a southern cluster, and, finally, one cluster with the outlier population SM (Fig 1B). The hierarchical AMOVA showed a stronger differentiation among BAPS clusters than among populations within clusters (Table 4). Contrary to other clusters, in the southern cluster the haplotype H11 is present (Figs 1 and 3). In the central cluster, the haplotype H6 is shared among all populations. The northern cluster had the lowest haplotypic diversity, with only 4 haplotypes and the PG population was fixed for H10 (Table 3).

**Table 4:** Analysis of molecular variance (AMOVA) of the *A*. *unedo* populations. Among all populations (a,c) and among the groups defined by the BAPS analysis (b,d) (hierarchical AMOVA). Distances between haplotypes were measured as number of different alleles (IAM) (a,b) and as number of repeat units for each microsatellite for the i-th locus (SMM) (c,d). SS = sum of squared deviations, d.f. = degrees of freedom, and P = probability of obtaining a more extreme value by chance alone.

Source of Variation	d.f.	SS	Variance Components	% of total variance	Φ statistics^a^	P
**(a) All populations**						
Among pops	14	39.60	0.09	27.97	Φst = 0.28	<0.0001
Within pops	436	97.30	0.22	72.03		
Total	450	136.90	0.31			
**(b) Hierarchical AMOVA**						
Among BAPS clusters	3	29.89	0.09	27.01	Φct = 0.27	<0.001
Among pops within clusters	11	9.71	0.02	6.53	Φsc = 0.09	<0.0001
Within pops	436	97.30	0.22	66.46	Φst = 0.33	<0.0001
Total	450	136.90	0.33			
**(c) All populations**						
Among pops	14	353.609	0.81	47.14	Φst = 0.47	<0.0001
Within pops	436	396.005	0.91	52.86		
Total	450	749.614	1.72			
**(d) Hierarchical AMOVA**						
Among BAPS clusters	3	310.689	0.99534	49.69	Φct = 0.50	<0.005
Among pops within clusters	11	42.920	0.09953	4.97	Φsc = 0.10	<0.0001
Within pops	436	396.005	0.90827	45.34	Φst = 0.55	<0.0001
Total	450	749.614				

^a^ Fixation indices computed in the Arlequin software [44].

Finally, the Mantel tests showed an absence of correlation between genetic and geographic distances with both mutation models and thus a lack of isolation by distance pattern. Considering all the populations, the G_ST_ = 0.29±0.11 was among population differentiation based on unordered alleles and the N_ST_ = 0.28±0.11 was based on ordered alleles. The observed N_ST_ was not significantly different from the observed G_ST_, showing absence of a phylogeographic structure.

## Discussion

### Spatial distribution of genetic diversity

There is evidence about two possible refugial areas for *A*. *unedo* in Portugal during the LGM, although it is worth noting that continuous pollen records in the region are missing [47] and that *A*. *unedo* is a low pollen producer often underrepresented in the pollen diagrams [48]. Nevertheless, Mateus & Queiroz [49] found palaeobotanical evidence of scrublands dominated by *Quercus coccifera* L., including *A*. *unedo* and other thermophiles shrubs, during the Middle Holocene in the southern region. Santiso *et al*. [5] also postulated a glacial refugium for the species in North Africa-Atlantic Iberia during the LGM. In addition, fossil charcoal records from central Portugal (Estremadura region) support the presence of Mediterranean taxa, including *A*. *unedo*, during the last glacial period [50]. According to these authors, survival of the thermophilous species in central Portugal during the LGM was possible due to the Atlantic Ocean proximity, enough protection from northerly winds and good solar exposure, and average temperatures and precipitations comparable to today. Therefore, it seems likely that most of these species expanded from central Portugal during climate amelioration and that a rapid reforestation occurred after the LGM [51]. In the application of the “refugia within refugia” model to the Iberian Peninsula, the paradigm of Iberia as a single refugium during Pleistocene glacial maxima was challenged, supporting instead the presence of several Iberian refugia for a range of flora and fauna [4]. Thus, combining already available knowledge [12] with our results, which are based on a more intensive genetic sampling of *A*. *unedo* in this area, the hypothesis of the presence of different refugia cannot be excluded. Two well-differentiated genetic clusters were found, one in central and another in southern Portugal. In both clusters, there are populations displaying high genetic diversity (e.g. EC and BV in the southern cluster; G, AV, ON, PS and SF in the central cluster) and some private haplotypes (e.g. high-frequency haplotype H6 is present only in the central and northern cluster). Such findings contrast with the hypothesis of a common postglacial origin of *A*. *unedo* populations in Portugal.

There are several points about the recent evolutionary history of *A*. *unedo* at its westernmost distribution limit that need to be clarified, and for which the use of genetic markers characterized by higher mutation rates (e.g. nuclear microsatellites) would be helpful. We observed an absence of phylogeographic structure and IBD patterns in our study that could be explained by not having considered the genetic structure found in the analysis. Nevertheless, when tests were repeated only on the central cluster, the only one with sufficient data to obtain meaningful results, the lack of phylogeographic structure and IBD was confirmed (results not shown). Overall, this points towards low levels of gene flow among populations probably due to limitations in the seed dispersal by birds. Whether limited gene flow may increase the effect of genetic drift in erasing IBD patterns, depends on overall gene exchanges among populations and, therefore, on both pollen and seed gene flow [52]. This could be disentangled by assessing both the genetic connectivity through pollen at different spatial scales and by the seed-to-pollen gene flow ratio in *A*. *unedo* to understand the impact of genetic drift on the spatial distribution of genetic diversity found in the present study.

Another important aspect to be addressed is the effect of contemporary factors on the distribution of genetic variation, with suitable markers. The occurrence of wildfires in the region potentially drives to abrupt changes in population size. Wildfires help in keeping the canopy open, a key factor for *A*. *unedo* survival and reproduction that prefers open-canopy environments. However if fires are too frequent or intense, the impact on population genetic diversity can be negative, as it is probably the case for the PG population (suffering on average wildfires with 5.7 years interval, since 1975) [53]. We should take into consideration that between 1975–2005, the total area burnt in Portugal was 3,353,000 ha, equivalent to 38% of the country, with a mean annual percent of burn area of 1.2% [22], yet with large inter-annual variability (see S1 Fig). Indeed, increase in fire frequency is seen as a major threat, since short inter-fire periods may exhaust soil-stored and fire-stimulated seed banks, reducing both the effective population size and genetic diversity [54] and eventually increasing the probability of extinction of forest tree populations.

### Design of conservation and provenance regions

In the studied region *A*. *unedo* is, currently, undergoing an intense domestication process, with the commercialisation of improved genetic material (clones) to produce new plantations and the introduction of genetic material through seed from unknown origin [11,17]. Domestication together with growing climate change will affect the species’ natural resources. Moreover, the use of vegetative propagation could lead to genetic erosion and inbreeding problems [55], this last effect might also affect the production of fruits, which is the interesting economic product.

Populations are not similar in their capacity to adapt to varying environmental conditions and genetic information can ensure a better use of the available resources by maximizing the evolutionary contribution of the set of populations to conserve [38]. Diversity alone, regardless of the way it is measured, is not a sufficient criterion to identify populations that deserve higher priority for conservation, and when different populations are chosen for conservation, redundancy should be avoided. For conservation purposes the uniqueness of a population, in terms of its allelic composition, may be at least as important as its diversity level, as advised by Petit *et al*. [38]. To select *A*. *unedo* candidate populations for conservation, besides the genetic diversity parameters, we used the contribution of each population to the total diversity partitioned into two components. The first was related to the level of diversity of the population and the second to its ‘uniqueness’, *i*.*e*., its divergence from the other populations [38].

We found that the SM population, representing a single-population genetic cluster, was the most differentiated, with a high contribution to divergence, but not to diversity levels. We suggest including this population in a conservation programme for *A*. *unedo* in Portugal since rare haplotypes will be conserved from a putative remnant population. The southern cluster EC population should be also considered as a potential gene reserve, due to its high genetic diversity and haplotypic richness, and for harbouring a private haplotype as SM. This population has the highest contribution to the total haplotypic richness in both the differentiation and diversity components. Similarly, the ON and AV populations, from the central cluster, should also be considered in conservation programs. The first population show the second highest values of genetic diversity and number of haplotypes, besides the average genetic distance among individuals, and the later has the highest haplotypic richness and number of private haplotypes in this cluster. Conservation should include the i) possible impact of very frequent wildfires in the genetic diversity, and the ii) impact of the species domestication at the region scale. In this region, maritime pine was a paradigmatic example of domestication impact in the species genetic structure: the use of seeds from unknown origin has completely blurred it and affected the species’ forest resources [56], and this should be avoided in the strawberry tree case.

Considering the species’ genetic improvement, the breeding population used in improvement program, including the clones used for deployment, should mirror the diversity and the haplotypic richness of the species in the region, and care should be taken to monitor the breeding generations ahead. This could be done by comparing the haplotypic and genetic diversity of the breeding population with the current study populations’ haplotypic and genetic diversity and by using additional markers, such as nuclear microsatellites and single nucleotide polymorphisms [57,58].

The information retrieved in the current study will also be helpful to design provenance regions aiming at collecting reproductive material for plantations. Those provenance regions should be built based on: i) genetic clustering results, and ii) ecological attributes, including wildfire information [59]. In order to further capture adaptive information, there would be a need for more genetic information, principally about adaptive traits and the genes underlying these traits important both for species preservation and genetic improvement. Direct information about adaptive variation in controlled environments could be done by installing genetic tests, including populations’ representative of the clusters detected in the current study. Provenance tests provide information about geographic patters of adaptive genetic variation, which can further help in defining the gene conservation populations. Additionally, they can direct the delineation of breeding zones and seed transfer guidelines, assuring that well adapted genotypes are deployed in artificial regeneration programs [59,60].

## Supporting information

S1 Fig Development of the percentage of burned area (sum of the burned areas/area of the country) and number of wildfires, per year, in the Southern Mediterranean countries, Portugal, Spain, France, Italy and Greece over a 30-year period (1980–2005). (A) Percentage of burned area development. (B) Number of wildfires development. European Commission (2007) Forest Fires in Europe 2006. European Commission, Joint Research Centre. Institute for Environment and Sustainability, Report 7. Luxembourg.
(TIF)

S2 Fig Sampled populations. Map highlighting the populations’ sampled (black dots) and the *A*. *unedo* stand distribution according to the 2006 Portuguese National Forest Inventory: http://www.icnf.pt/portal/florestas/ifn (orange triangles).
(TIF)

S1 Table The absolute (counts) and relative (frequency) frequencies of the fifteen detected haplotypes. Haplotype refers to four chloroplast microsatellites composition respectively: AU1, AU2, AU4 and AU7.
(DOCX)
