# Supplementary figures and images for: Genetic diversity and divergence at the Arbutus unedo L. (Ericaceae) westernmost distribution limit
Source: PLoS One. 2017 Apr 6;12(4):e0175239. doi: 10.1371/journal.pone.0175239 (PMC5383270; doi:10.1371/journal.pone.0175239)

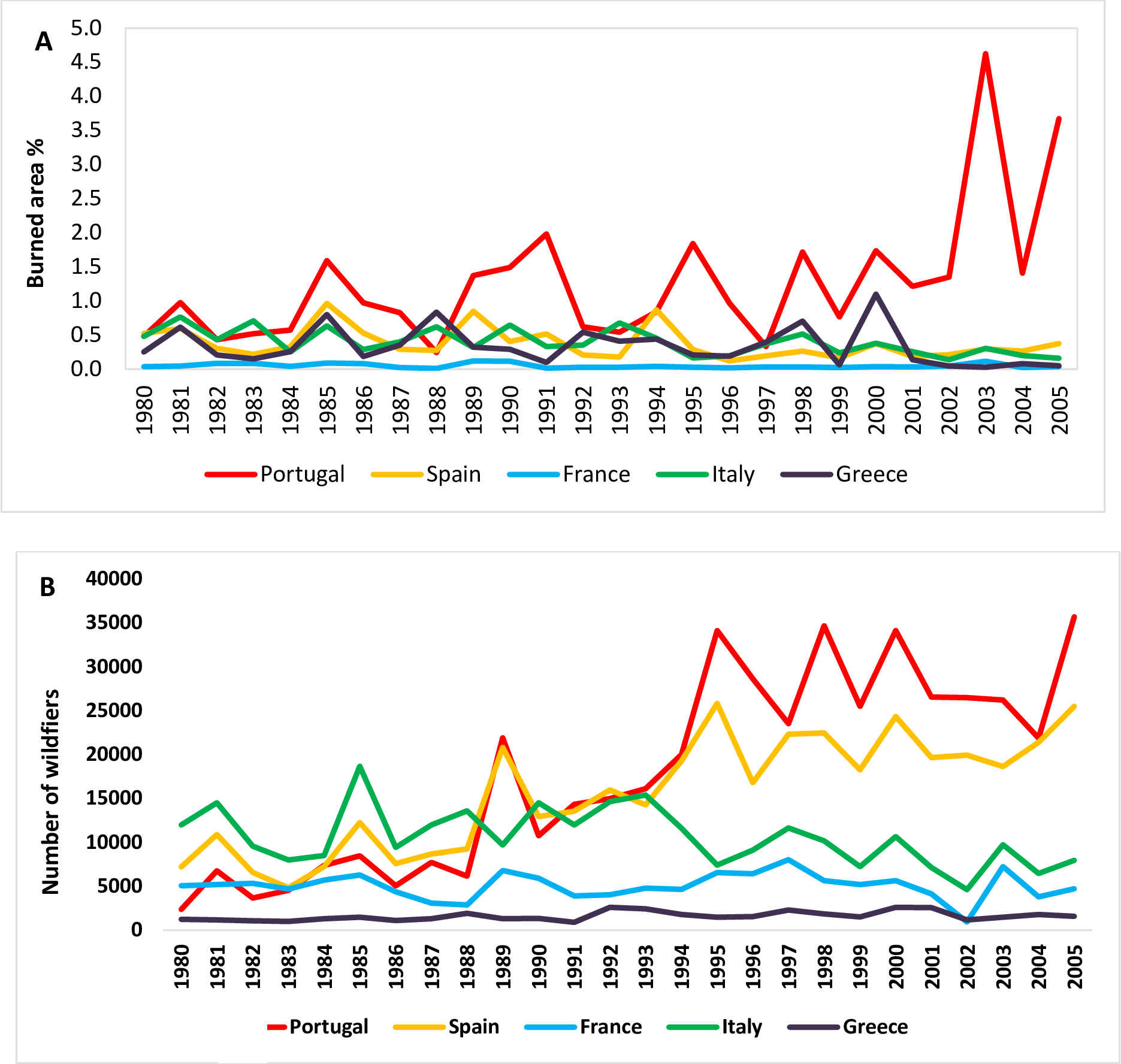

Supplement: S1 Fig — (A) Percentage of burned area development. (B) Number of wildfires development. European Commission (2007) Forest Fires in Europe 2006. European Commission, Joint Research Centre. Institute for Environment and Sustainability, Report 7. Luxembourg. (TIF) [file pone.0175239.s001.tif]

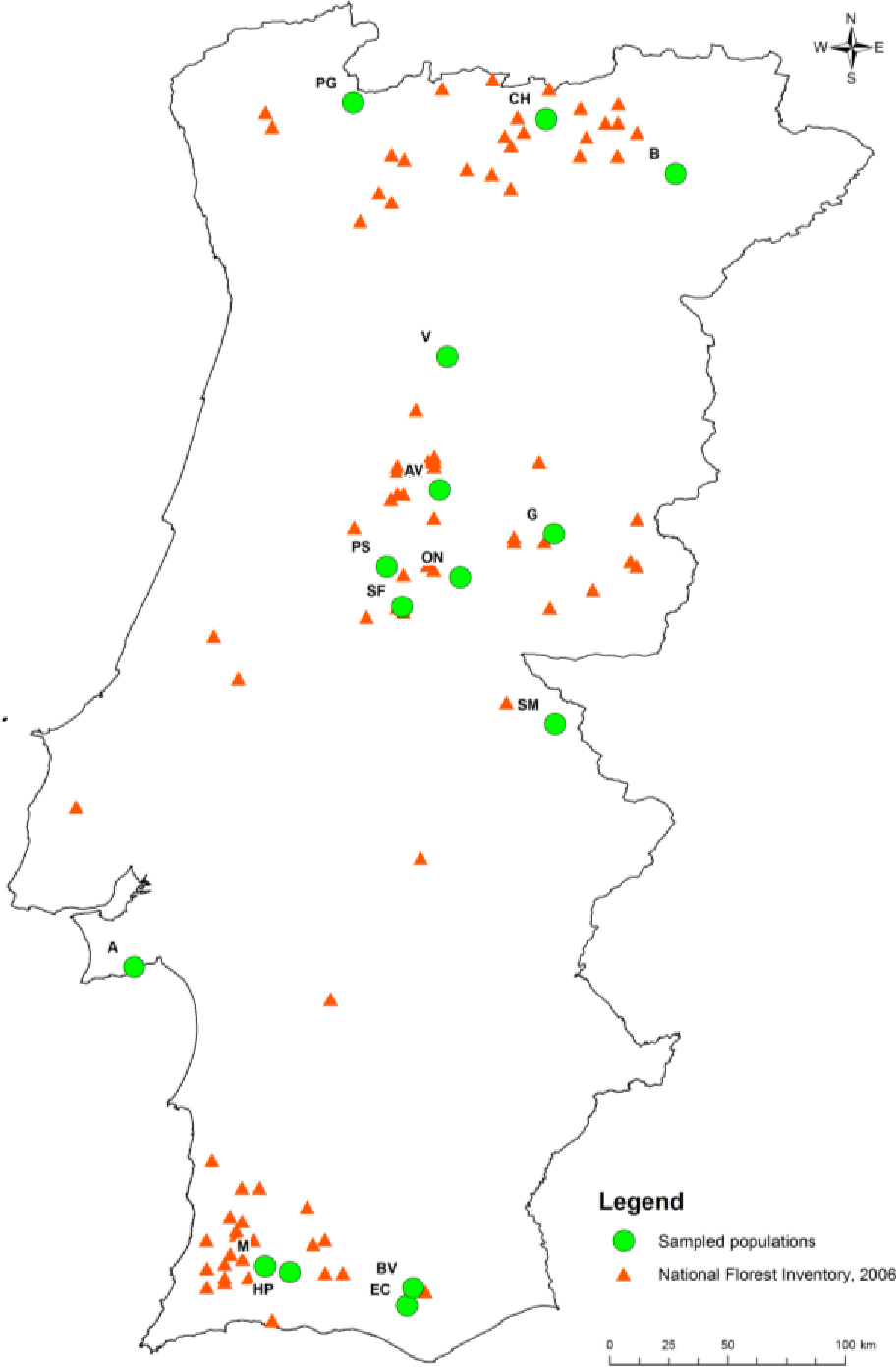

Supplement: S2 Fig — Map highlighting the populations’ sampled (black dots) and the A. unedo stand distribution according to the 2006 Portuguese National Forest Inventory: http://www.icnf.pt/portal/florestas/ifn (orange triangles). (TIF) [file pone.0175239.s002.tif]
