# Supplementary material for: Genetic diversity and divergence at the Arbutus unedo L. (Ericaceae) westernmost distribution limit
Source: PLoS One. 2017 Apr 6;12(4):e0175239. doi: 10.1371/journal.pone.0175239 (PMC5383270; doi:10.1371/journal.pone.0175239)
Supplement: S1 Table — Haplotype refers to four chloroplast microsatellites composition respectively: AU1, AU2, AU4 and AU7. (DOCX) [file pone.0175239.s003.docx]

| **Label** | **Haplotype**  AU1/AU2/AU4/AU7 | **Counts** | **Frequency** |
| --- | --- | --- | --- |
| H1 | 306/282/385/370 | 1 | 0.002 |
| H2 | 306/283/385/372 | 7 | 0.016 |
| H3 | 306/285/385/372 | 6 | 0.013 |
| H4 | 306/294/385/372 | 1 | 0.002 |
| H5 | 307/278/385/372 | 3 | 0.007 |
| H6 | 307/279/385/372 | 70 | 0.155 |
| H7 | 307/282/385/372 | 6 | 0.013 |
| H8 | 307/283/383/372 | 1 | 0.002 |
| H9 | 307/283/385/370 | 2 | 0.004 |
| H10 | 307/283/385/372 | 291 | 0.645 |
| H11 | 307/284/385/372 | 13 | 0.029 |
| H12 | 307/285/385/372 | 3 | 0.007 |
| H13 | 307/294/385/372 | 44 | 0.098 |
| H14 | 308/279/385/372 | 1 | 0.002 |
| H15 | 308/283/385/372 | 2 | 0.004 |
